# Supplementary material for: Human IPSC-Derived Model to Study Myelin Disruption
Source: Int J Mol Sci. 2021 Aug 31;22(17):9473. doi: 10.3390/ijms22179473 (PMC8430601; doi:10.3390/ijms22179473)
Supplement: Supplementary file 1 [file ijms-22-09473-s001.zip › ijms-1339545-supplementary-original/supplementary material 2.pdf]

2.1.- Environmental exposures linked to impairment of central nervous system myelination during development

**Compound Name(s)**	**Chemical Formula**	**Molecular weight (g/mol)**	**CAS number**	**Supplier**	**Product Number**	**Conc.**	**Solubility**			**Myelin DNT Ref.**
							Conc.	***Solvent***	***Ref.***	
Bisphenol A (BPA)	$C_{15}H_{16}O_2$	228.291	80-05-7	Sigma Aldrich	239658-50G	-	0.298 g/L at 25°C	H_2O	Sigma Aldrich	Xu et al. 2016<br>Tiwari et al. 2015<br>Seiwa et al. 2004
2,4-Dichlorophenoxyacetic acid (2,4-D)	$C_8H_6Cl_2O_3$	221.033	94-75-7	Sigma Aldrich	D7299-100G	-	100 mg/ml	95% EtOH	Sigma Aldrich	Konjuh et al. 2008
							30 mg/ml	EtOH, DMF, DMSO	Cayman Chem.	
							540 ppm at 20°C	H_2O	Pub-Chem	
Tris(1,3-dichloro-2-propyl) phosphate (TDCPP)	$C_9H_{15}Cl_6O_4P$	430.889	13674-87-8	Sigma Aldrich	32951-100MG	-	0.0181 g/L at 20°C	H_2O	Sigma Aldrich	Wang et al. 2015
Bis(cyclohexanone)oxaldihydrazone (Cuprizone)	$C_{14}H_{22}N_4O_2$	278.356	370-81-0	Sigma Aldrich	14690-100G	-	$9.0x10^{-7}$ mg/L at 20°C	H_2O	Pub-Chem	Wang et al. 2013<br>Abe et at. 2015<br>Cammer, 1999
Sodium metavanadate	NaO_3V	121.928	13718-26-8	Sigma Aldrich	72060-100G					Mustapha et al. 2014
Nicotine	$C_{10}H_{14}N_2$	162.236	54-11-5	Sigma Aldrich	613215-1EA	20 mg/mL in DMSO				Cao et al. 2013
Pentabromodiphenyl oxide (DE-71)	$C_{12}H_5Br_5O$	564.691	32534-81-9	Great Lakes Chemical Corp.		20.1 mM	25 mM	DMSO	Giordano et al. 2008	Chen et al. 2012
Methylmercury	CH_3Hg^+	215.627	22967-92-6							Padhi et al. 2012

2.2.- Cytotoxicity and Myelin DNT

Compound (Abbreviation)	Cytotoxicity in vitro			Myelin DNT			
	Conc.	*notes*	*Ref.*	*Conc. / Dose*	*in vivo/in vitro; species*	*DNT Effect(s)*	*Ref.*
Bisphenol A (BPA)	100 µM	in vitro; rat oligospheres; highest non-cytotoxic conc.	Tiwari et al. 2015	0.1 mg/L H_20	in vivo; rat	Disrupted OL ER signaling; enduring OL loss	Xu et al. 2016
	100 µM	In vitro; mouse OPCs; almost 100% cells viable	Seiwa et al. 2004	100 µM	in vitro; rat OL spheres	Decreased viability, proliferation, and differentiation of OPCs; altered immunoreactivity of fluoromyelin, MBP, and myelin-associated glycoprotein; altered expression and protein levels of myelination-regulating genes; caused myelin structural changes	Tiwari et al. 2015
				100 µM	in vitro; mouse	Inhibited differentiation of OPCs; inhibited MBP expression	Seiwa et al. 2004
2,4-Dichlorophenoxyacetic acid (2,4-D)	1000 µM	Cytotoxicity limit	EPA ToxCast	70 mg/kgBW (DMSO)	in vivo; rat	Decreased myelin proteins (CNPase, PLP, and MBP); disrupted myelin layers and reduced number of sheets (electonmicrograph)<br>*also no effect on myelin structure was found in DMSO treated group	Konjuh et al. 2008
tris(1,3-dichloro-2-propyl) phosphate (TDCPP)	6.71 µM	Cytotoxicity limit	EPA ToxCast	0.001% (v/v) DMSO	in vivo; zebrafish	Detected in F1 eggs following parental exposure; MBP significantly downregulated in exposed F1 larvae	Wang et al. 2015
Bis(cyclohexanone) oxaldihydrazone (Cuprizone)				0.2% diet	in vivo; mouse	Demyelination in juvenile (3 weeks) and young-adult mice (6 weeks); reduction of myelin basic protein; loss of CC-1-positive mature oligodendrocytes	Wang et al. 2013
				0, 0.1 or 0.4% diet	in vivo; rat	Maternal exposure to 0.4% CPZ in the diet from gestational day 6 to day 21 decreases proliferative type-2 progenitor cells via endoplasmic reticulum stress-mediated apoptosis and inhibition of cholinergic signals to intermediate-stage progenitor cells following reduced oligodendrocyte production and suppression of the brain-derived neurotrophic factor signaling cascade	Abe et at. 2015
				25 µM (for 1 hour, each day 2-12 days)	in vitro; rat OL and mixed glial-cell cultures	Treatment with cuprizone produced a slight decrease in the total number of O4-positive oligodendrocytes; cuprizone consistently and significantly reduced the numbers of the O4-positive cells that would mature to the O4+/MBP+ stage	Cammer, 1999

Sodium metavanadate	300 μM	in vitro; mouse OL spheres; IC50	Mustapha et al. 2014	300 μM	in vitro; mouse OL spheres	Decreased expression of oligodendrocyte specific proteins: CNPase, OSP / Claudin	Mustapha et al. 2014
Nicotine				3 mg/kg BW	in vivo; rat	Significantly altered myelin gene expression, indicating that exposure disturbs OL development and myelin formation	Cao et al. 2013
Pentabromodiphenyl oxide (DE-71)	1000 μM	Cytotoxicity limit	EPA ToxCast	0.16, 0.8, 4.0 μg/L H_2O	in vivo; zebrafish	Residue of DE-71 was detected in F1 eggs upon parental exposure; Genes of central nervous system development (e.g., myelin basic protein, synapsin IIa, α1-tubulin) were significantly downregulated in larvae	Chen et al. 2012
	500 mg/mL	hippocampal neurons; 50% reduction in mitotic index	Giordano et al. 2008				
	2.2 μM	hippocampal neurons; IC50					
	8.9 μM	SK-N-SH neuroblastoma; MTT reduction; threshold	Yu et al. 2008				
	22.1 μM	SK-N-SH neuroblastoma; LDH release; threshold					
	6.4 μM	SK-N-SH neuroblastoma; apoptosis; PI staining; threshold					
Methylmercury				2 mg/kg BW	in vivo; rat	Reductions in total and individual MBP splice variant mRNA levels suggest that methylmercury-induced perturbation in MBP gene expression occurred due to decreased oligodendrocyte cell population	Padhi et al. 2012

OL = oligodendrocyte
NSC = neural stem cell
OPC = oligodendrocyte progenitor cell
OSP = oligodendrocyte specific protein
MPB = myelin basic protein
PLP = myelin proteolipid protein
CNPase = 2',3'-Cyclic-nucleotide 3'-phosphodiesterase

References

Giordano G, Kavanagh TJ, Costa LG. 2008. Neurotoxicity of a polybrominated diphenyl ether mixture (DE-71) in mouse neurons and astrocytes is modulated by intracellular glutathione levels. Toxicol Appl Pharmacol 232(2):161-168; doi: 10.1016/j.taap.2008.06.018 [doi].

Xu XB, Fan SJ, He Y, et al. Loss of hippocampal oligodendrocytes contributes to the deficit of contextual fear learning in adult rats experiencing early bisphenol A exposure. *Mol Neurobiol*. 2016.

Tiwari SK, Agarwal S, Chauhan LK, Mishra VN, Chaturvedi RK. Bisphenol-A impairs myelination potential during development in the hippocampus of the rat brain. *Mol Neurobiol*. 2015;51(3):1395-1416.

Seiwa C, Nakahara J, Komiyama T, Katsu Y, Iguchi T, Asou H. Bisphenol A exerts thyroid-hormone-like effects on mouse oligodendrocyte precursor cells. *Neuroendocrinology*. 2004;80(1):21-30.

Duffard R, Garcia G, Rosso S, et al. Central nervous system myelin deficit in rats exposed to 2,4-dichlorophenoxyacetic acid throughout lactation. *Neurotoxicol Teratol*. 1996;18(6):691-696.

Konjuh C, Garcia G, Lopez L, de Duffard AM, Brusco A, Duffard R. Neonatal hypomyelination by the herbicide 2,4-dichlorophenoxyacetic acid. chemical and ultrastructural studies in rats. *Toxicol Sci*. 2008;104(2):332-340.

Wang Q, Lai NL, Wang X, et al. Bioconcentration and transfer of the organophorous flame retardant 1,3-dichloro-2-propyl phosphate causes thyroid endocrine disruption and developmental neurotoxicity in zebrafish larvae. *Environ Sci Technol*. 2015;49(8):5123-5132.

Wang H, Li C, Wang H, et al. Cuprizone-induced demyelination in mice: Age-related vulnerability and exploratory behavior deficit. *Neurosci Bull*. 2013;29(2):251-259.

Chen L, Yu K, Huang C, et al. Prenatal transfer of polybrominated diphenyl ethers (PBDEs) results in developmental neurotoxicity in zebrafish larvae. *Environ Sci Technol*. 2012;46(17):9727-9734.

Mustapha O, Oke B, Offen N, Siren AL, Olopade J. Neurobehavioral and cytotoxic effects of vanadium during oligodendrocyte maturation: A protective role for erythropoietin. *Environ Toxicol Pharmacol*. 2014;38(1):98-111.

Cao J, Wang J, Dwyer JB, et al. Gestational nicotine exposure modifies myelin gene expression in the brains of adolescent rats with sex differences. *Transl Psychiatry*. 2013;3:e247.

Padhi BK, Pelletier G. Perturbation of myelin basic protein (mbp) splice variant expression in developing rat cerebellum following perinatal exposure to methylmercury. *Toxicol Lett*. 2012;213(3):374-380.

Yu K, He Y, Yeung LW, Lam PK, Wu RS, Zhou B. 2008. DE-71-induced apoptosis involving intracellular calcium and the bax-mitochondria-caspase protease pathway in human neuroblastoma cells in vitro. Toxicol Sci 104(2):341-351; doi: 10.1093/toxsci/kfn088 [doi].

Eskes C, Honegger P, Jones-Lepp T, Varner K, Matthieu JM, Monnet-Tschudi F. Neurotoxicity of dibutyltin in aggregating brain cell cultures. *Toxicol In Vitro*. 1999;13(4-5):555-560.

Chantong B, Kratschmar DV, Lister A, Odermatt A. Dibutyltin promotes oxidative stress and increases inflammatory mediators in BV-2 microglia cells. *Toxicol Lett*. 2014;230(2):177-187.

EPA ToxCast - https://actor.epa.gov/dashboard

PubChem - https://pubchem.ncbi.nlm.nih.gov
