# Supplementary material for: Human IPSC-Derived Model to Study Myelin Disruption
Source: Int J Mol Sci. 2021 Aug 31;22(17):9473. doi: 10.3390/ijms22179473 (PMC8430601; doi:10.3390/ijms22179473)
Supplement: Supplementary file 1 [file ijms-22-09473-s001.zip › ijms-1339545-supplementary-original/Supplementary materials 1. .pdf]

Neural progenitor cell mycoplasma testing report

grcf at Johns Hopkins
DNA Services

Laura Kasch-Semenza
Johns Hopkins University
2760 Light House Point East, Suite 201
Baltimore, Maryland 21224
Phone: 410-614-3830
Email: lkasch@jhmi.edu

JOHNS HOPKINS MEDICINE

Mycoplasma*Testing*Report*

*

Date:*July%24,%2017%
Customer:*Thomas%Hartung%
Institution:*Johns%Hopkins%University* * * * * * *
Person*requesting:*Shelly-Ann Odwin-DaCosta%
GeneSifter*ID*number:*138034%
%

Sample Annotation:

**Chip Number:**	30005498
**Well:**	A6
**Sample ID:**	1

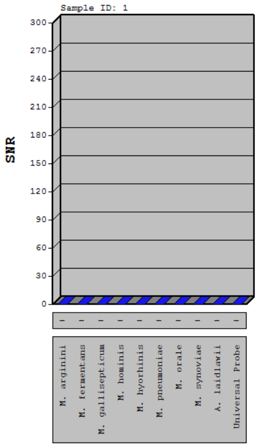

Bacterium	SNR	Result	Level
M. arginini	0	negative	-
M. fermentans	0	negative	-
M. gallisepticum	0	negative	-
M. hominis	0	negative	-
M. hyorhinis	0	negative	-
M. pneumoniae	0	negative	-
M. orale	0	negative	-
M. synoviae	0	negative	-
A. laidlawii	0	negative	-
Universal Probe	0	negative	-

%

GRCF services may only be used for research purposes. Any data or samples generated by the facility may not be used for human diagnostic or therapeutic use.

grcf at Johns Hopkins
DNA Services

Laura Kasch-Semenza
Johns Hopkins University
2760 Light House Point East, Suite 201
Baltimore, Maryland 21224
Phone: 410-614-3830
Email: lkasch@jhmi.edu

JOHNS HOPKINS MEDICINE

Mycoplasma*Testing*

*

The GRCF's mycoplasma test uses a PCR based MycoDtect™ kit from Greiner Bio-One North America, Inc. (Monroe, NC) to PCR amplify the 16S – 23S intergenic spacer region with a highly conserved fluorescent primer pair. The amplified fragments are hybridized to a MycoDtect™ DNA-array. A universal mycoplasma DNA-probe tests for the presence of all mycoplasma species, while species-specific probes detect nine of the most common mycoplasma. Each array is run with positive and negative controls in addition to internal DNA isolation and PCR controls for each cell line sample.

Procedure*

DNA is extracted from the cell culture cells and supernatant using a DNeasy Blood & Tissue kit automated on a QIAcube (Qiagen). A MycoDtect internal control is added to each sample prior to isolation to monitor the DNA extraction process and template use for the amplification. The DNA is polymerase chain amplified (PCR) for the 16S-23S rRNA intergenic spacer region of any mycoplasma species present using a highly conserved Cy5 fluorescent labeled primer pair. A PCR control within the PCR MasterMix allows for validation of the PCR. The labeled products are hybridized to complementary sequences on the MycoDtect chip. Each mycoplasma species, the universal probe, and internal and PCR controls are detected by five measuring points on the chip. Non-specifically bound probes are removed by washing. The bound and labeled probes are detected by stimulation with monochromatic light and analyzed using CheckReport software. Appropriate positive and negative controls were used.

The MycoDtect assay will detect both viable and non-viable mycoplasma, it will not distinguish between the two.

Sensitivity*

The limit of detection determined by the manufacturer with the universal probe for the nine specific species identified is 1 colony forming unit (CFU), except M. fermentas, which is 10 CFU.

Greiner Bio-One has tested MycoDtect™ and determined the universal probe to detect the following forty Mollicutes species: A. axanthum, A. laidlawii, A. modicum, A. morum, A. oculi, A. vituli, M. alkalescens, M. arginini, M.arthritidis, M. bovigenitalium, M. bovirhinis, M. bovis, M. bovoculi, M. buccale, M. californicum,M.canadense, M. canis, M. eqhirhinis, M. faucium,M. fermentans, M. flocculare, M. gallinaceum, M.gallinarum, M. gallisepticum, M. genitalium, M. glycophilium, M. hominis, M. hyopharyngis, M.hyopneumoniae, M. hyorhinis, M. hyosynoviae, M. orale, M. pirum, M. pneumoniae, M. pulmonis, M.salivarium, M. synoviae, S. citri, S. kunkelii, U. diversum, U. urealyticum.

Below are species-specific mycoplasmas detected with MycoDtect, the natural host, and likely sources of contamination.

Species	Host of mycoplasma species	Source of contamination
M. orale	human	laboratory personnel
M. fermentans	human	laboratory personnel
M. hominis	human	laboratory personnel
M. pneumoniae	human	laboratory personnel
M. arginini	bovine	serum
A. laidlawii	bovine	serum
M. hyorhinis	swine	serum
M. gallisepticum	poultry	media
M. synoviae	poultry	media

*

Neural progenitor cell karyotyping report

Case name: CLG-19146

Cell Line Name: NPC GEO 1 p16

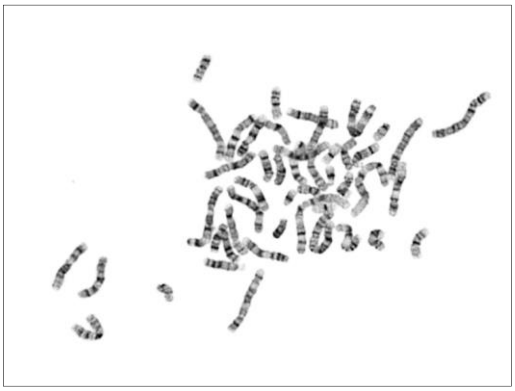

Result: 46,XY,add(18)(q21)

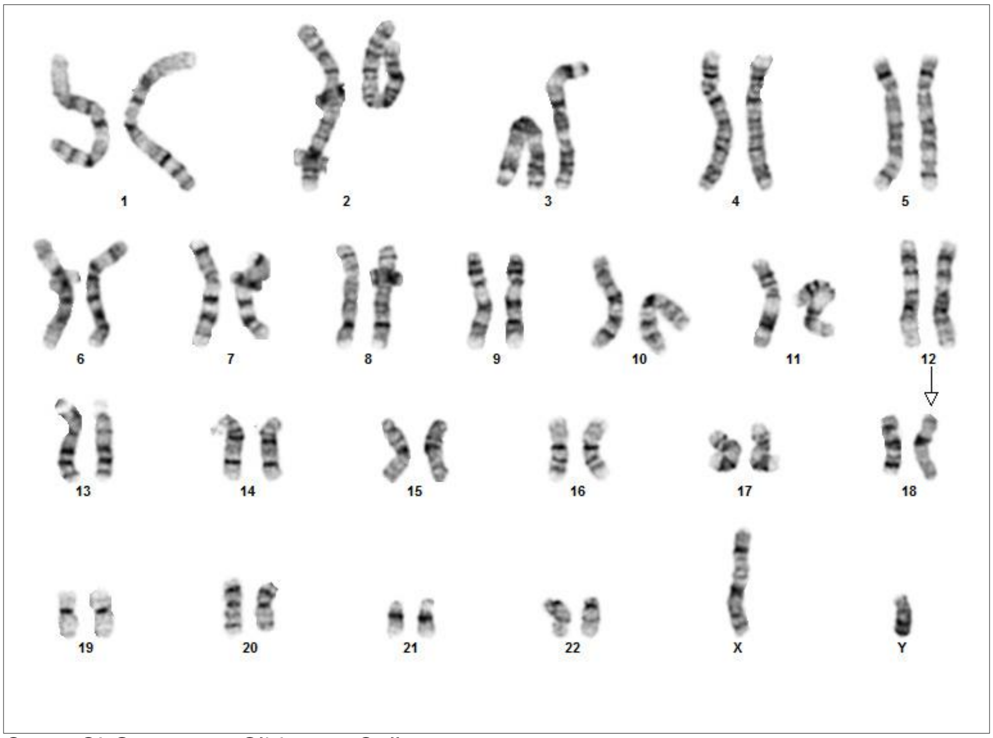

Case: CLG-19146 Slide: 1 Cell: 16

Cell Line Characterization

Cell Line ID: NPC GEO1
Passage #: 16
Specimen Type: Human Neuronal Precursor Culture
Indication for Study: Routine Culture QC

Lab #: CLG-19146
PI: Not Specified
Contact Person: Ruth Brady
Email: rbrady1@jhu.edu
Address:
Johns Hopkins University
615 N. Wolfe St W7032
Baltimore, Maryland 21205

Test Code: 100
Account #: NA
PO #: NA

Date Received: 2/19/15
Date Reported: 3/2/15
Time in Culture: 1 day

Additional copies sent to:

Banding Technique: GTL **Band Resolution:** Fair
Metaphases Counted: 20 Analyzed: 7 Karyotyped: 4

RESULTS: 46,XY,add(18)(q21)[17] ABNORMAL Human Male Karyotype

Non-clonal Aberrations: 46,XY,del(2)(q32.2),add(18)(q21)
45,XY,-14,add(18)(q21)
45,XY,-16,add(18)(q21)

INTERPRETATION:

Cytogenetic analysis was performed on twenty G-banded metaphase cells from human cell line NPC GEO1 p16 and all twenty cells demonstrated an addition of unknown genetic material to the long-arm of chromosome 18 at band 18q21. Three of these cells also demonstrated non-clonal chromosome aberrations (listed above) which are most likely artifacts of culture.
