# Supplementary material for: Human IPSC-Derived Model to Study Myelin Disruption
Source: Int J Mol Sci. 2021 Aug 31;22(17):9473. doi: 10.3390/ijms22179473 (PMC8430601; doi:10.3390/ijms22179473)
Supplement: Supplementary file 1 [file ijms-22-09473-s001.zip › ijms-1339545-supplementary-original/Supplementary table 1.pdf]

Supplementary table 1.
Environmental exposures linked to impairment of central nervous system myelination during development.

Compound Name(s)	Chemical Formula	Molecular weight (g/mol)	CAS number	Supplier	Product Number	Conc.	Solubility			Myelin DNT Ref.
							Conc.	***Solvent***	***Ref.***	
Bisphenol A (BPA)	$C_{15}H_{16}O_2$	228.291	80-05-7	Sigma Aldrich	239658-50G	-	0.298 g/L at 25°C	H_2O	Sigma Aldrich	(Xu et al., 2017; Tiwari et al., 2015; Seiwa et al., 2004)
2,4-Dichlorophenoxyacetic acid (2,4-D)	$C_8H_6Cl_2O_3$	221.033	94-75-7	Sigma Aldrich	D7299-100G	-	100 mg/ml	95% EtOH	Sigma Aldrich	(Konjuh et al., 2008)
							30 mg/ml	EtOH, DMF, DMSO	Cayman Chem.	
							540 ppm at 20°C	H_2O	Pub-Chem	
Tris(1,3-dichloro-2-propyl) phosphate (TDCPP)	$C_9H_{15}Cl_6O_4P$	430.889	13674-87-8	Sigma Aldrich	32951-100MG	-	0.0181 g/L at 20°C	H_2O	Sigma Aldrich	(Wang et al., 2015)
Bis(cyclohexanone)oxaldihydrazone (Cuprizone)	$C_{14}H_{22}N_4O_2$	278.356	370-81-0	Sigma Aldrich	14690-100G	-	$9.0x10^{-7}$ mg/L at 20°C	H_2O	Pub-Chem	(Wang et al., 2013; Cammer, 1999) Abe et at. 2015
Sodium metavanadate	NaO_3V	121.928	13718-26-8	Sigma Aldrich	72060-100G					(Mustapha et al., 2014)
Nicotine	$C_{10}H_{14}N_2$	162.236	54-11-5	Sigma Aldrich	613215-1EA	20 mg/mL in DMSO				(Cao et al., 2013)
Pentabromodiphenyl oxide (DE-71)	$C_{12}H_5Br_5O$	564.691	32534-81-9	Great Lakes Chemical Corp.		20.1 mM	25 mM	DMSO	Giordano et al. 2008	(Chen et al., 2012)
Methylmercury	CH_3Hg^+	215.627	22967-92-6							(Padhi and Pelletier, 2012)

Cammer, W. (1999). The neurotoxicant, cuprizone, retards the differentiation of oligodendrocytes in vitro. *Journal of the Neurological Sciences 168*, 116-120. doi:Doi 10.1016/S0022-510x(99)00181-1

Cao, J., Wang, J., Dwyer, J. B. et al. (2013). Gestational nicotine exposure modifies myelin gene expression in the brains of adolescent rats with sex differences. *Transl Psychiatry 3*, e247. doi:10.1038/tp.2013.21

Chen, L., Huang, C., Hu, C. et al. (2012). Acute exposure to de-71: Effects on locomotor behavior and developmental neurotoxicity in zebrafish larvae. *Environ Toxicol Chem 31*, 2338-2344. doi:10.1002/etc.1958

Konjuh, C., Garcia, G., Lopez, L. et al. (2008). Neonatal hypomyelination by the herbicide 2,4-dichlorophenoxyacetic acid. Chemical and ultrastructural studies in rats. *Toxicological Sciences 104*, 332-340. doi:10.1093/toxsci/kfn085

Mustapha, O., Oke, B., Offen, N. et al. (2014). Neurobehavioral and cytotoxic effects of vanadium during oligodendrocyte maturation: A protective role for erythropoietin. *Environmental Toxicology and Pharmacology 38*, 98-111. doi:10.1016/j.etap.2014.05.001

Padhi, B. K. and Pelletier, G. (2012). Perturbation of myelin basic protein (mbp) splice variant expression in developing rat cerebellum following perinatal exposure to methylmercury. *Toxicology Letters 213*, 374-380. doi:10.1016/j.toxlet.2012.07.011

Seiwa, C., Nakahara, J., Komiyama, T. et al. (2004). Bisphenol a exerts thyroid-hormone-like effects on mouse oligodendrocyte precursor cells. *Neuroendocrinology 80*, 21-30. doi:10.1159/000080663

Tiwari, S. K., Agarwal, S., Chauhan, L. K. S. et al. (2015). Bisphenol-a impairs myelination potential during development in the hippocampus of the rat brain. *Molecular Neurobiology 51*, 1395-1416. doi:10.1007/s12035-014-8817-3

Wang, H. K., Li, C. R., Wang, H. Z. et al. (2013). Cuprizone-induced demyelination in mice: Age-related vulnerability and exploratory behavior deficit. *Neuroscience Bulletin 29*, 251-259. doi:10.1007/s12264-013-1323-1

Wang, Q., Lai, N. L., Wang, X. et al. (2015). Bioconcentration and transfer of the organophorous flame retardant 1,3-dichloro-2-propyl phosphate causes thyroid endocrine disruption and developmental neurotoxicity in zebrafish larvae. *Environ Sci Technol 49*, 5123-5132. doi:10.1021/acs.est.5b00558

Xu, X. B., Fan, S. J., He, Y. et al. (2017). Loss of hippocampal oligodendrocytes contributes to the deficit of contextual fear learning in adult rats experiencing early bisphenol a exposure. *Mol Neurobiol 54*, 4524-4536. doi:10.1007/s12035-016-0003-3
