# Supplementary figures and images for: Human IPSC-Derived Model to Study Myelin Disruption
Source: Int J Mol Sci. 2021 Aug 31;22(17):9473. doi: 10.3390/ijms22179473 (PMC8430601; doi:10.3390/ijms22179473)

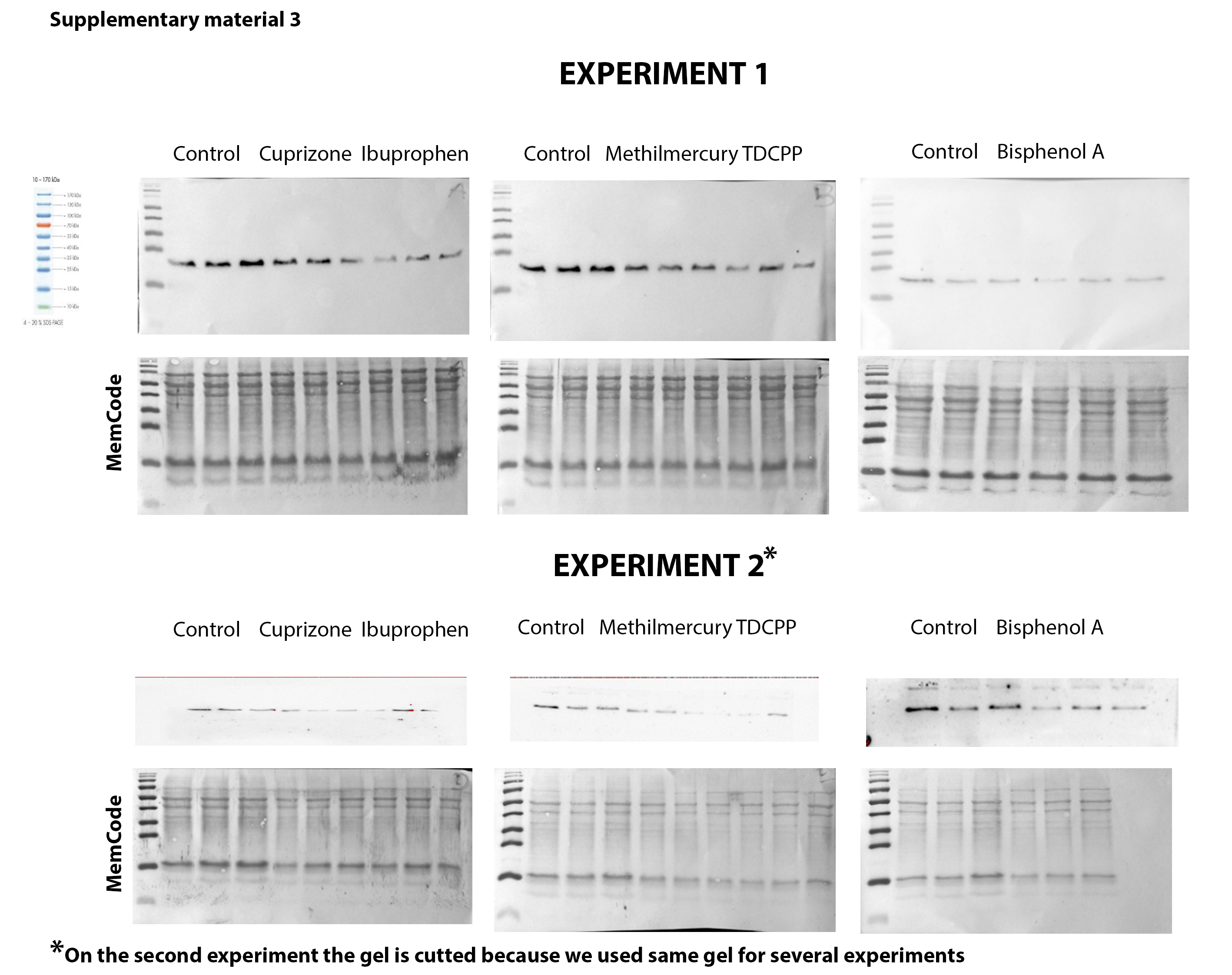

Supplement: Supplementary file 1 [file ijms-22-09473-s001.zip › ijms-1339545-supplementary-original/supplementary material 3.jpg]
